# Metallo-β-Lactamase Inhibitors Inspired on Snapshots from the Catalytic Mechanism

**DOI:** 10.3390/biom10060854

**Published:** 2020-06-03

**Authors:** Antonela R. Palacios, María-Agustina Rossi, Graciela S. Mahler, Alejandro J. Vila

**Affiliations:** 1 Instituto de Biología Molecular y Celular de Rosario (IBR, CONICET-UNR), Ocampo and Esmeralda, S2002LRK Rosario, Argentina; palacios@ibr-conicet.gov.ar (A.R.P.); rossi@ibr-conicet.gov.ar (M.-A.-R.); 2 Laboratorio de Química Farmacéutica, Facultad de Química, Universidad de la Republica (UdelaR), Montevideo 11800, Uruguay; gmahler@fq.edu.uy; 3 Área Biofísica, Facultad de Ciencias Bioquímicas y Farmacéuticas, Universidad Nacional de Rosario, S2002LRK Rosario, Argentina

**Keywords:** metallo-β-lactamases, mechanism-based inhibitors, antibiotic resistance, reaction mechanism

## Abstract

β-Lactam antibiotics are the most widely prescribed antibacterial drugs due to their low toxicity and broad spectrum. Their action is counteracted by different resistance mechanisms developed by bacteria. Among them, the most common strategy is the expression of β-lactamases, enzymes that hydrolyze the amide bond present in all β-lactam compounds. There are several inhibitors against serine-β-lactamases (SBLs). Metallo-β-lactamases (MBLs) are Zn(II)-dependent enzymes able to hydrolyze most β-lactam antibiotics, and no clinically useful inhibitors against them have yet been approved. Despite their large structural diversity, MBLs have a common catalytic mechanism with similar reaction species. Here, we describe a number of MBL inhibitors that mimic different species formed during the hydrolysis process: substrate, transition state, intermediate, or product. Recent advances in the development of boron-based and thiol-based inhibitors are discussed in the light of the mechanism of MBLs. We also discuss the use of chelators as a possible strategy, since Zn(II) ions are essential for substrate binding and catalysis.

## 1. Antibiotic Resistance Mediated by β-Lactamases

### 1.1. β-Lactams Are the Most Clinically Used Antibiotics

Antibiotic resistance has led to a global health crisis, as resistant infectious bacteria are becoming more widespread every day all over the world. According to the latest report of the Centers for Disease Control and Prevention (CDC), a post-antibiotic era has begun, where the successful medical approaches to combat bacterial infections are being threatened [1]. Epidemiologic studies disclose a direct correlation between antibiotic consumption and the emergence or dissemination of resistant bacteria [2]. Antibiotics can eradicate susceptible microorganisms, but at the same time, they also introduce an evolutionary pressure that drives the natural selection of the resistant ones, which can reproduce and disseminate [3,4,5]. These events are exacerbated by the misuse and abusive use of antibiotics that push the limits of bacterial resistance [6].

**Figure 1:**
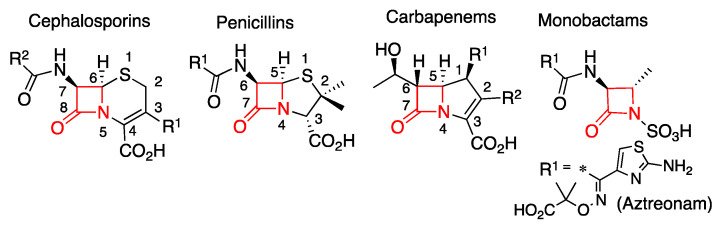
General scaffold of clinical β-lactam antibiotics with their respective atom numbering. The β-lactam ring is shown in red.

β-Lactam antibiotics are the most frequently prescribed and best-selling antimicrobial drugs (Figure 1) [7,8]. Their success is due to their broad-spectrum of activity and their favorable safety profile. These compounds share, as a common active group, a four-membered cyclic amide called a β-lactam ring (Figure 1). Its similarity to the D-Ala D-Ala terminal dimer of peptidoglycan triggers a cross recognition by Penicillin-binding proteins (PBPs), which consequently weakens the bacterial membrane and causes cell lysis [9,10]. The most clinically used β-lactam antibiotics (cephalosporins, penicillins, and carbapenems) have an additional five- or six-membered ring. Instead, only one monocyclic β-lactam compound is clinically used, the monobactam aztreonam (Figure 1) [11,12]. These basic structures can be decorated with different substituents resulting in derivatives with diverse properties such as acidic tolerance (allowing its oral administration) and a broad antimicrobial spectrum.

In cephalosporins, the β-lactam ring is fused to a six-membered sulfur-containing dihydrothiazine ring (Figure 1) [10]. These are the most widely prescribed and diverse class of β-lactams, since they have two variable substituents R^1^ and R^2^ at position C3 and C9, respectively. The cephalosporin derivatives (mainly semi-synthetic) are grouped into five generations according to their antimicrobial activity [13]. The reactivity of some clinically relevant cephalosporins is incremented by the presence of good leaving groups in position R^1^ that may be detached during the reaction, giving rise to an exocyclic double bond. Penicillins contain a five-membered thiazolidine ring (Figure 1) and were the first discovered β-lactam antibiotics [11,14]. Carbapenems are the latest generation of β-lactams. Their β-lactam ring is fused to a five-membered ring but contains only carbon atoms and a double bond on C2-C3 (Figure 1). These compounds have a huge clinical relevance due to their broad antimicrobial spectrum, good tolerance and the reduced number of resistance mechanisms against them compared to penicillins and cephalosporins. For these reasons, these antibiotics are reserved as last resort treatments for resistant bacterial infections [15]. All approved carbapenems are administered as intra-venous drugs, but recently tebipenem was developed as the first oral compound from this family, currently in phase 3 clinical trials [16,17].

The evolution of different resistance mechanisms has put in danger even the last resort treatments, as many bacteria are now resistant to all clinically used drugs [18]. Furthermore, in recent years, there has been a significant decrease on the discovery of new antibiotics. The rapid emergence of antibiotic resistance decreases the clinical lifespan of these drugs and, as a consequence, the development of new compounds implies a great effort that does not guarantee proper revenues [19]. Teixobactin, discovered in 2015 and currently in clinical trials, was the first new antibiotic developed in 27 years [20,21]. The promising new β-lactam cefiderocol is a siderophore-modified cephalosporin active against carbapenem-resistant Gram-negative bacteria and was approved by the Food and Drug Administration of the United States (FDA), Department of Health and Human Services in 2019 [22,23,24,25]. Alternative treatments resort to the design and development of compounds that are able to inhibit resistance mechanisms and can be co-administered with already known antibiotics, aiming to prolong their clinically useful lifetime [8]. Despite many advances, this still remains a challenging field, especially for the treatment of multidrug-resistant bacterial infections.

### 1.2. β-Lactamases Are the Main Resistance Mechanism against β-Lactam Antibiotics

**Figure 2:**
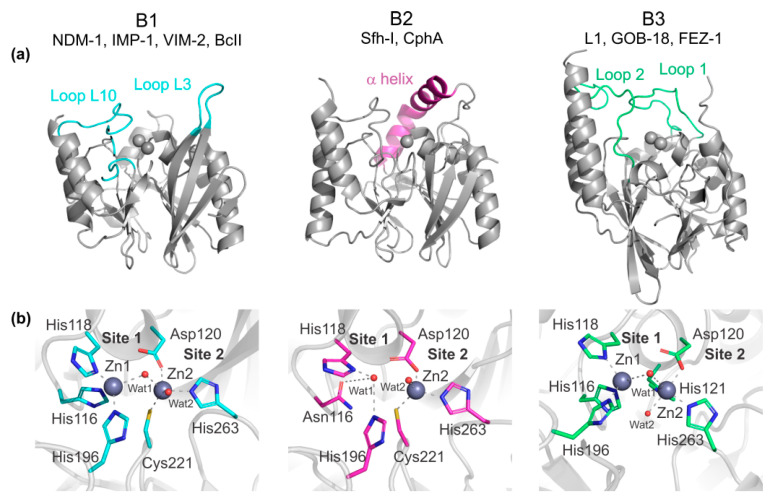
Representative structures of the three metallo-β-lactamases (MBLs) subclasses: B1 NDM-1 (PDB 3spu), B2 Sfh-I (PDB 3sd9), and B3 L1 (PDB 1sml). (**a**) Overall protein structures. Loops and helices involved in substrate interactions are shown in color. (**b**) Active sites. Zn(II) ions are shown as grey spheres and water/OH molecules as red spheres. Zn(II) interactions are shown as dashed lines and metal ligands are shown in color. Residues are numbered according to the standard MBL numbering scheme. For the B2 enzyme Sfh-I, the active mono-Zn(II) form is shown.

There are several mechanisms by which bacteria become resistant to β-lactam antibiotics [26]. The most widespread resistance mechanism (both in Gram-negative and Gram-positive bacteria) is the expression of hydrolytic enzymes named β-lactamases [27,28]. These enzymes catalyze the cleavage of the amide bond of β-lactams (Figure 1), rendering the antibiotics ineffective against their targets [26]. More than 4000 β-lactamases have been identified to date, and based on their structural features, they are divided into four groups from A to D (Ambler classification) [29,30]. Classes A, C, and D are serine-β-lactamases (SBLs) [31], which employ an activated Ser residue as nucleophile to hydrolyze the β-lactam antibiotics [32,33]. Among them, class A β-lactamases were the first ones to be discovered and have been thoroughly studied. On the other hand, class B are Zn(II)-dependent hydrolases (Figure 2), generally known as metallo-β-lactamases (MBLs) [31]. These enzymes do not share any structural, mechanistic or sequence homology with SBLs or PBPs, indicating an independent evolutionary origin [34,35,36]. While different SBLs have specific substrate profiles, and only a few of them are active against carbapenems, MBLs hydrolyze all β-lactams antibiotics (with the exception of aztreonam and the recently approved cefiderocol [37]) and their clinical relevance is due to their potent carbapenemase activity [34,35,36]. In contrast to SBLs, there are no clinically approved inhibitors of MBLs [28].

Based on a molecular criterion, MBLs are divided into three subclasses (B1, B2, and B3) that differ by the active site residues, metal content requirement, and substrate profile (Figure 2) [35]. B1 enzymes need two Zn(II) ions to be fully active and have a broad substrate spectrum. The most clinically relevant MBLs, such as the NDM [38], VIM [39], IMP [40], SPM [41], and CcrA [42] families, belong to this subclass. The genes coding for most of these enzymes were disseminated worldwide on mobile genetic elements, aggravating the geographical spread of resistance [18]. Multiple variants of these enzymes can be found in Gram-negative pathogens such as *Pseudomonas*, *Klebsiella*, and *Acinetobacter* species, among others. BcII, the β-lactamase of the non-pathogenic bacterium *Bacillus cereus*, also belongs to the B1 subclass and was the first MBL described [43,44]. Indeed, this enzyme was originally reported in 1966, long before MBLs became widespread in the clinical setting, and was employed to perform most of the seminal studies [45,46,47,48,49]. B2 MBLs are chromosomally encoded enzymes active with only one Zn(II) ion and are specific carbapenemases, showing poor hydrolytic capacities against penicillins and cephalosporins [50,51,52,53,54]. CphA from *Aeromonas hydrophila* [53], Sfh-I from *Serratia fonticola* [55] and ImiS from *Aeromonas sobria* [52] are representative members of this group. B3 enzymes can be either active with one or two Zn(II) ions, have a broad substrate spectrum and, remarkably, only 9 residues are conserved among these enzymes and the B1 and B2 MBLs [56]. GOB from *Elizabethkingia meningoseptica* [56], L1 from *Stenotrophomonas maltophilia* [57,58] and FEZ from *Legionella gormanii* [59] are the most studied B3 MBLs.

The characteristic fold of all MBLs is an αβ/βα sandwich, with two central β sheets and five solvent-exposed α helices (Figure 2a). The metal active site is situated on a wide groove delimitated by the interface of two domains. In B1 enzymes, the active site is flanked by two functionally relevant loops named L3 and L10 (Figure 2a). L3 is mobile and has been showed to play several roles in substrate binding and catalysis through enzyme-dependent hydrophobic interactions [60,61,62,63,64,65]. In B2 enzymes, an elongated α helix (α3) closes over the active site groove providing hydrophobic interactions that form a hydrophobic wall [53,54]. The orientation of α3 in B2 enzymes limits the accessibility of bulky substrates and has been proposed to be responsible for their restricted substrate spectrum [53,54,66]. In B3 enzymes, two mobile loops named 1 and 2 delimitate the active site and are also involved in substrate recognition [56,57,67]. The flexibility of mobile loops flanking the already wide active sites in B1 and B3 enzymes facilitates binding and hydrolysis of different substrates and adaptation to different evolutionary challenges [68]. A common strategy to design new antibiotics escaping the action of SBLs has relied in the inclusion of bulky substituents in the classic β-lactam scaffolds [8]. However, the plasticity of the active sites of MBLs allows them to accommodate compounds with larger substituents and invalidates that strategy [69,70,71].

MBLs have two metal binding sites, site 1 and site 2 (Figure 2b), whose features differ among the distinct subclasses. In B1 enzymes, two Zn(II) ions are bound to the active site, with a bridging water/hydroxide molecule (Wat1). The Zn(II) on the site 1 (Zn1) is coordinated to His116, His118, His196, and Wat1 (standard MBL numbering scheme [72,73] used throughout), and the second ion (Zn2) is coordinated to Asp120, Cys221, His263, Wat1, and a second water molecule (Wat2) (Figure 2b) [46,49,74]. In B3 enzymes, the site 1 from B1 β-lactamases is preserved, while site 2 involves residues Asp120, His121, and His263 as metal ligands (Figure 2b). B3 enzymes from the GOB family can be fully active with only one metal ion located in site 2 [56,75]. Finally, B2 enzymes are active with only one Zn(II) ion bound to a ligand set similar to that from the Zn2 site of B1 subclass [53,54,66]. A His166Asn substitution, present in all B2 enzymes, changes the conformation of the typical Zn1 site in such a way that binding of a second metal equivalent inactivates these β-lactamases [76]. In addition to these active site features, there are several residues involved in substrate/inhibitor binding [28,77] such as a positively charged residue in position 224 (typically a Lys residue), 233 (Asn), and 228 (an Arg in the VIM family) [78], whose interactions will be described later in this review.

## 2. Mechanism-Based β-Lactamase Inhibitors

### 2.1. Crucial Differences on the Mechanism of β-Lactamases

**Figure 3:**
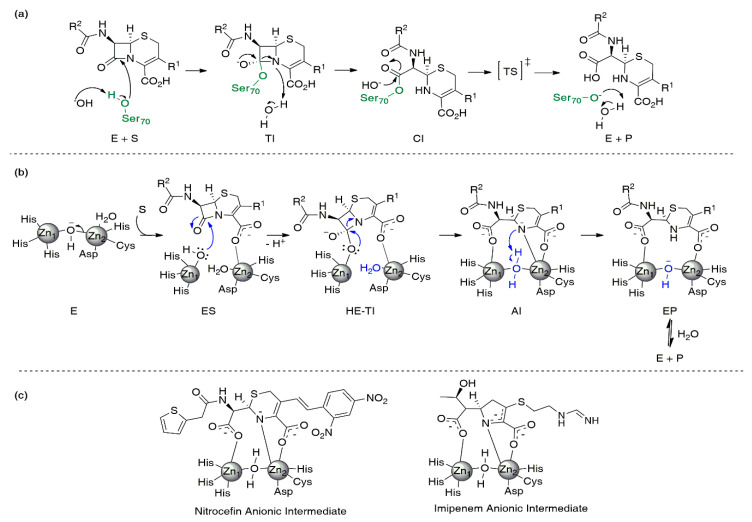
Reaction mechanism of serine-β-lactamases (SBLs) and MBLs. General reaction mechanisms for cephalosporin hydrolysis by (**a**) SBLs and (**b**) MBLs. (**c**) Reaction intermediates formed during nitrocefin and imipenem hydrolysis by MBLs. The following abbreviations were employed: Enzyme (E), Substrate (S), Tetrahedral Intermediate (TI), Covalent Intermediate (CI), Product (P), Enzyme:Substrate Complex (ES), High-Energy Tetrahedral Intermediate (HE-TI), Anionic Intermediate (AI), and Enzyme:Product Complex (EP).

SBLs and MBLs catalyze the irreversible hydrolysis of the β-lactam ring by means of substantially different reaction mechanisms (Figure 3). SBLs employ an essential Ser residue for catalysis (Figure 3a), as the result of an evolution from the mechanism of action of PBPs [32,33]. An activated hydroxyl group from this residue is responsible for the nucleophilic attack on the amide bond of the β-lactam. This step produces a reaction intermediate known as the tetrahedral intermediate, based on the sp^3^ hybridization of the carbon atom derived from the β-lactam. This species involves a covalent bond between the former carbonyl carbon of the β-lactam and the oxygen of the active site Ser residue (Figure 3a) [79,80]. The tetrahedral intermediate has a negative charge that is stabilized by interactions with a positively charged cleft on the active site, known as the oxyanion hole. Then, the cleavage of the amide bond gives rise to the formation of a covalent acyl-enzyme intermediate (Figure 3a) [28]. The last reaction step is the protonation and cleavage of the covalent bond that maintains the hydrolyzed product bound to the enzyme. This deacylation event is the rate-limiting step of the mechanism [81,82].

Early proposals for the reaction mechanism of MBLs were inspired by the mechanism of SBLs [83,84]. However, the lack of the catalytic Ser residue and the oxyanion hole makes it impossible to extrapolate this mechanism to MBLs [85]. Currently, it is widely accepted that there are two main steps during the reaction that have been detected experimentally: the nucleophilic attack on the carbonyl and the protonation of the N atom (Figure 3b) [85,86,87,88]. Nevertheless, within this general scheme, there have been several controversial issues such as the accumulation of reaction intermediates [86], the identity of the nucleophile and the proton donor [83,89,90], and the specific function of each metal ion during the process [90,91,92,93].

The Zn(II) ions are key pieces of the reaction mechanism of MBLs. Substrate positioning in the active site, nucleophile activation, the stabilization of the different species formed during the mechanism, and the positioning of the proton donor strongly depend on these metal ions (Figure 3b) [87]. In Zn(II) hydrolases (such as glyoxalase II [94], carbonic anhydrase [95], and carboxypeptidase A [96]), the metal ion lowers the pKa of a bound water molecule, thus eliciting a functional hydroxide group in the active site, promoting the nucleophilic attack. For B1 and B3 MBLs, it has been proposed that Zn1 accomplishes a similar function [83,86,87,97]. However, since Zn1 is absent in B2 MBLs, Zn2 is the only preserved structural feature in all MBLs and plays an essential role in positioning and stabilizing different species during the reaction [87] (see below).

In spite of structural differences, there are several common features of the reaction mechanism of MBLs from the three subclasses (Figure 3b). Upon substrate binding, the Michaelis complex involves interactions with the metal site and surrounding residues. The nucleophilic attack is produced by a water/hydroxide molecule activated by binding to the metal ion (B1 and B3) or by hydrogen-bonding interactions (B2). Unlike SBLs, the tetrahedral intermediate formed before the cleavage of the β-lactam ring is energetically close to the related transition states and has not been trapped. Instead, experimental evidence demonstrates that anionic intermediates are formed and stabilized during the hydrolysis of cephalosporins and carbapenems (Figure 3b,c) [47,65,86,87,89,97,98,99,100,101,102,103,104]. These intermediates are bound to the active site by electrostatic interactions and lack a tetrahedral carbon, since cleavage of the C-N bond in the β-lactam ring has already taken place (Figure 3b). The negative charge is stabilized by a strong interaction with Zn2 and is delocalized within the structure of the hydrolyzed antibiotic [87,100,105,106]. Then, protonation of these species (the rate-limiting step) gives rise to product formation, with the ultimate release and recovery of the free enzyme.

### 2.2. Mechanism-Based Inhibitors of SBLs 

The development of β-lactamase inhibitors involves the design of an efficient inhibitory and non-toxic molecule that, in combination with a β-lactam antibiotic, must display similar pharmacokinetic properties for its prescription. Several SBLs inhibitors are available since the seminal introduction of clavulanic acid (a compound of the family of clavams) in 1984 combined with amoxicillin and ticarcillin [107]. Soon after, two penicillanic acid sulfones were developed: sulbactam and tazobactam. Combinations of these compounds with different penicillins and cephalosporins are used to treat infections caused by bacteria expressing many class A SBLs [8,28,108]. These drugs cannot inhibit class B, C, and D β-lactamases, nor class A carbapenemases such as KPC. Their mechanism of action is based on the reaction mechanism of SBLs, being susceptible to hydrolysis by the catalytic Ser residue. This leads to an irreversible inactivation of the enzyme due to an inefficient deacylation step that results in covalent binding of these compounds to the active Ser residue [80,82]. These compounds are all conceived as mechanism-based suicide substrates.

Avibactam is a recently developed mechanism-based β-lactamase inhibitor whose functional group is a diazabicyclo[3.2.1]octanone (DBO) [109,110]. Unlike the previously described compounds, avibactam does not contain a β-lactam ring but it is also susceptible to hydrolysis, and can acylate SBLs in a reversible manner [109,110]. The ceftazidime-avibactam combination is approved for clinical use since 2015 and is active against SBLs from all subclasses, including those with carbapenemase activity. Despite this compound not being active against MBLs, the combination of avibactam with aztreonam is active against *Enterobacterales* expressing both SBLs and MBLs (since aztreonam can escape the action of MBLs). This formulation is currently on phase 3 clinical trials [8].

Boron-based inhibitors have been studied since the 1980s [111], but vaborbactam was the first clinical boron-based SBL inhibitor, approved by the FDA in 2017 in combination with meropenem [8]. This inhibitor is active against class A and class C SBLs and is ineffective against class D enzymes and MBLs. Vaborbactam is a competitive inhibitor that forms a reversible covalent bond with the active Ser residue [112]. Other DBOs and boron-inhibitors are in clinical trials, with relebactam (a DBO) being the most advanced [8,28,109].

### 2.3. Challenges for the Design of a Broad-Spectrum MBL Inhibitor

None of the approved SBL inhibitors are active against MBLs, and the vast knowledge gained on SBL inhibition cannot be directly applied to the design of MBL inhibitors [8]. This is mainly due to the previously described differences between the catalytic mechanisms of these two classes of β-lactamases [85,87], the identity of the attacking nucleophile (a Ser hydroxyl group versus an activated water/hydroxide molecule that results in the lack of a covalent intermediate in MBL-based hydrolysis) and the nature of the intermediate species accumulated in each case (Figure 3). Furthermore, the active site topologies of SBLs and MBLs are completely different. SBLs have a narrow and deep catalytic site located on the interface of two domains, where the active Ser residue is buried [28]. Instead, the active site in MBLs is positioned in a shallow groove in which the Zn(II) ions are in its base and are solvent exposed. An additional difficulty resides on the large structural diversity of MBLs in comparison to SBLs, which involves (1) different active site topologies (Figure 2), (2) low sequence homology among active site residues, and (3) different Zn(II) stoichiometries [35,36], as discussed in previous sections.

The literature accounts for a large number of efforts aimed to design MBL inhibitors applying a wide variety of strategies. The rational design has been limited by the structural variability of MBLs. Several compounds have been discovered based on the established experience of inhibition of zinc enzymes (such as the use of hydroxamates, thiol groups, and sulfonamide compounds, among others) but none of them has made it to the clinic yet. Chemical libraries and fragment-based screenings (with the concomitant molecule optimization) [113,114], virtual screening employing libraries of natural products or synthetic compounds [115,116], and extracts from plants or microorganisms [117,118,119] led to potential inhibitors. Many of these compounds were highly effective against some particular MBLs but a cross class inhibition was rarely obtained. A broad-spectrum inhibitor could be designed based on the interactions of the different mechanistic species that, as we detailed before, are conserved in the reaction mechanism of all MBLs.

Numerous excellent review articles have covered many of these efforts [8,120,121,122,123,124,125,126]. In this case, we aim to provide a different perspective, focusing on MBL inhibitors inspired by the interactions of the different species formed during catalysis with the enzymes. We will first analyze inhibitors based on substrate structures or tetrahedral transition state interactions. Then, we will discuss those based on the stable anionic intermediate or Enzyme:Product (EP) structures. Finally, we will briefly analyze the use of chelators on MBL inhibition, since they remove the essential Zn(II) ions.

## 3. MBL Inhibitors Based on Substrate Structures

### 3.1. Substrate Binding and Initial Transition State

Substrate binding, the first step in the reaction mechanism, is often accompanied by conformational changes. Despite many efforts, there are no crystal structures of the Michaelis complex (ES) of a β-lactam compound bound to an MBL. In the case of bi-metallated MBLs, evidence of conformational changes upon substrate binding was obtained from stopped-flow fluorescence experiments in the enzymes L1 and BcII [97,127]. These experiments have also shown that the Zn(II) ions are essential for substrate binding, playing a key electrostatic anchoring role. Changes in the coordination sphere of the metal site on the Michaelis complexes were assessed by rapid-freeze quench mixing experiments coupled to X-ray absorption spectroscopy [87,128,129] and also by stopped-flow and rapid freeze quenching experiments in Co(II)-substituted enzymes [86,87,130,131], further supporting this notion. In addition to substrate-metal interactions, several works have shown that active site L3 and L10 in B1 enzymes play an important role in substrate binding [60,132,133,134,135,136,137]. Based on these experimental results, in the availability of different crystal structures of EP complexes [69,101,135,138,139,140] and many docking studies [49,105,106,141,142], a general substrate binding mode has been proposed.

**Figure 4:**
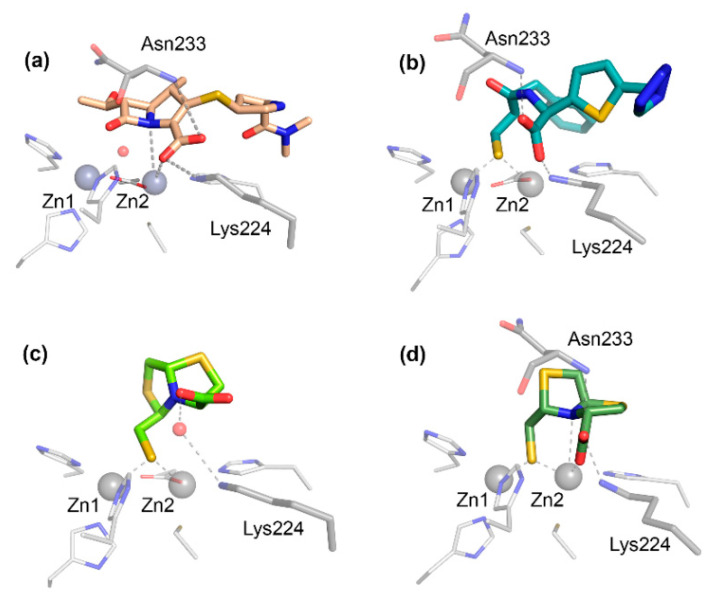
Binding modes of substrate mimic inhibitors. (**a**) Modeled structure of meropenem bound to NDM-1; crystal complexes of (**b**) mercaptocarboxylate **1**:IMP-1 (PDB 1dd6) [148], (**c**) L-CS319 L-**6**:NDM-1 (PDB 4u4l) [149], and (**d**) D-CS319 D-**6**:IMP-1 (PDB 5ev8) [150]. Hydrogen bonds and metal ligands interactions are drawn with dashed lines.

The carboxylate group present in all bicyclic β-lactam compounds (Figure 1) provides the main driving force for binding, coordinating directly to Zn2 and interacting with a positively charged residue in position 224, generally a Lys in B1 MBLs (Figure 4a), except in the case of VIM enzymes, in which the interaction takes place with residue 228. This productive binding mode positions the β-lactam ring close to the bridging hydroxide of bi-Zn(II) enzymes, favoring the nucleophilic attack (Figure 3b). Additional interactions with the main chain nitrogen of Asn233 are conserved (Figure 4a). The different substituents of the antibiotics can be accommodated in the active site groove, where very few specific interactions have been identified. Aztreonam binds to MBLs in an unproductive binding mode in which a direct interaction of the sulfonate group with Zn2 leaves the β-lactam group positioned far away from the attacking nucleophile, resulting in no hydrolysis [143].

B2 MBLs are exclusive carbapenemases and show their maximum efficiency on their mono-metallated form (Figure 2) [34,50]. The substrate binding mode and catalytic mechanism are based on the crystallographic structure of CphA with hydrolyzed biapenem [53], on docking experiments [54,144], and on studies of the intrinsic fluorescence of ImiS that revealed the presence of conformational changes during substrate binding and product release [145]. The first of these events involves the coordination of the carboxylate to the metal ion and secondary interactions with Lys224 and Val67. The main difference with bi-metallated MBLs is that the attacking water molecule is located on the metal-vacant site 1 (Figure 2) and its activation is facilitated by hydrogen bonding interactions with His118 and Asp120 [53,54,144,146]. This leads to a less potent nucleophile, resulting in more stable ES complexes than those formed on bi-metallated enzymes, allowing their study by spectroscopic techniques [87]. Binding of a second metal ion in the putative site 1 has been shown to displace the nucleophile, giving rise to an inactive enzyme [76]. The cleavage of the amide bond is also promoted by the interaction of the N atom with the Zn(II) ion [87,144].

In all MBLs, the nucleophilic attack has been proposed to give rise to a high-energy tetrahedral intermediate (HE-TI, Figure 3b) [147] before cleavage of the C-N bond. This high energy state partially resembles the tetrahedral intermediate proposed for SBLs (TI, Figure 3a) but it has not been characterized experimentally. The main feature is the formation of the sp^3^ carbon near the Zn1 site, while the interaction of the carboxylate group with the Zn2 site is assumed to be preserved.

### 3.2. Substrate Mimic Inhibitors

As the formation of the ES complex involves specific substrate-enzyme interactions, it is expected that molecules mimicking either the interaction modes or the structures will be efficient inhibitors. Furthermore, since most MBLs are broad-spectrum enzymes able to bind most β-lactam substrates, this strategy enables the design of compounds that can potentially bind many MBLs. In this section, we will review several compounds that were designed based on the β-lactam antibiotic structure.

**Figure 5:**
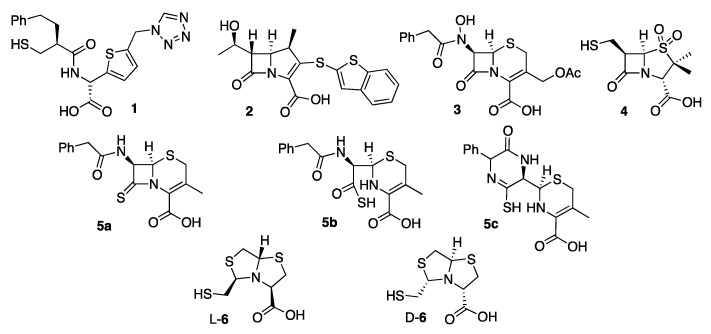
MBL substrate mimic inhibitors.

The first MBL substrate mimic inhibitor reported was the mercaptocarboxylate **1** (Figure 5) [148]. This compound contains some of the chemical moieties present in benzylpenicillin: a linear amide, the carboxylate and an aromatic moiety that mimics the R^1^ chain. It is further decorated with a thiol, a well-known zinc binding group. This compound displayed an inhibition of IMP-1 and L1 in the nM range (Table 1) (nM range covers inhibition constants (*K_i_*) or half-maximal inhibitory concentration (IC_50_) lower than 90 nM, used throughout). In the case of IMP-1, the thiol binds the metal center, bridging the two Zn(II) ions and displacing the catalytic water present in the resting state enzyme. The rest of the molecule shows interactions with key residues such as Asn233 and Lys224 (Figure 4b) and hydrophobic contacts with L3 [148]. Despite these substrate mimicking features, inhibition and binding are driven by the strong Zn(II)-thiolate interaction, while the rest of the inhibitor resides within the active site forming hydrogen bonds that do not resemble those of the substrates.

Different approaches have been used to design bicyclic substrate mimics. One includes the addition of different groups to the bicyclic core of β-lactams. A screening on a library of 1β-methylcarbapenem derivatives allowed the identification of J-110,441 **2** (Figure 5) [151]. A benzothiophene group at C2 led to strong inhibition potencies with a *K_i_* values in the low-μM range for B1 and B3 MBLs (Table 1) (the low-μM range covers potencies ranging from 9 μM to 0.1 μM, used throughout) and SBLs from classes A and C. The incorporation of spacers, the replacement using the same side chain but substituted at position 3 or modification of some methines for amides led to poor inhibition potencies. **2** potentiated the sensitivity of clinical isolates to imipenem or ceftazidime [151].

Buynak et al. employed the cephalosporin scaffold by exploring the replacement of N-H at C7 by N-OH, forming a reverse hydroxamate which can bind the Zn(II) ions at the active site. The best-performing derivative **3** (Figure 5) showed an IC_50_ value in the low-μM range against B1 enzymes (Table 1) [152].

Dual SBL and MBL inhibitors were designed within the penicillin scaffold by including modifications that generate a stable acyl-enzyme complex aimed to pursue SBL inhibition and a thiol group targeting MBLs [153]. Among them, compound **4** (Figure 5) showed an IC_50_ value in the low-μM range against BcII, L1, TEM-1 and P99 (Table 1). In combination with piperacillin, **4** reduced the MICs of different bacterial strains producing MBLs, including *E. coli*-IMP-1 and *P. aeruginosa* with VIM or SPM-1 [153].

Another substrate mimicking strategy has relied on reducing the reactivity of the β-lactam antibiotics towards hydrolysis while maintaining the binding features to the active site. In 8-thioxocephalosporins, the β-lactam amide is substituted by a thioamide, affecting the reactivity, charge distribution and acidity of the compounds [154]. The 8-thioxocephalosporin **5a** (Figure 5) was indeed poorly hydrolyzed by BcII, as predicted, and showed a weak inhibitory activity (Table 1). Instead, the thioacid **5b** (product of hydrolysis, Figure 5) gave rise to a stronger inhibitory effect on BcII (*K_i_* = 96 μM) [154,155]. The thioxocephalexin in solution is prone to an intramolecular aminolysis leading to a mono-thioxo-piperazinedione **5c** (Figure 5) which inhibits BcII with a greater potency (*K_i_* = 29 μM) [152,154]. 

The bisthiazolidine scaffold (*cf.* L-**6** and D-**6** in Figure 5) was designed as a substrate mimic inspired by the structure of penicillins but lacks the amide moiety and with a thiol as the metal binding group. These inhibitors were successful against MBLs from the three subclasses, including the B1 enzymes BcII, NDM-1, IMP-1 and VIM-2 [78], the B2 enzyme Sfh-I, and the B3 enzymes L1 and GOB-18 (B3) [149,150]. The stereochemistry of the chiral centers had an impact on the inhibitory power in the case of Sfh-I, due to its more restricted active site. Binding of the bisthiazolidines to binuclear B1 and B3 enzymes was governed by the interaction of the thiol group with the metal center, while the carboxylate group shows distinct interactions with conserved residues such as Asn233 and Lys224 in the B1 subclass (Figure 4c,d). As a result, these compounds show a versatile binding mode and can be accommodated in the active site of MBLs. The most potent inhibitor L-**6** (Figure 5 and Table 1) restored the β-lactam antimicrobial effect in a variety of MBL-producing clinical strains. No toxic effects were observed against eukaryotic cells and L-**6** did not inhibit glyoxalase II, a human metalloenzyme with an MBL-related protein fold.

### 3.3. Transition State Analogues

**Figure 6:**
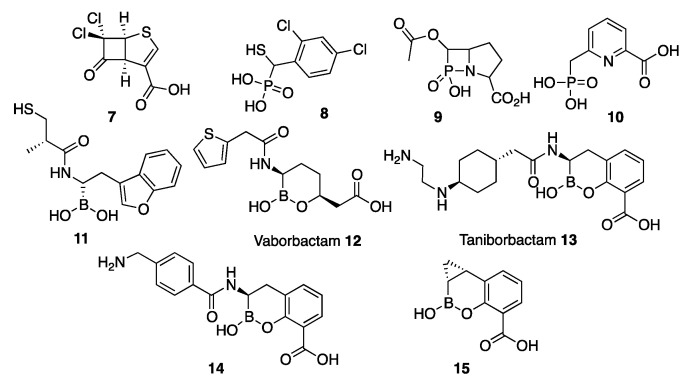
Inhibitors designed as transition state analogues.

**Figure 7:**
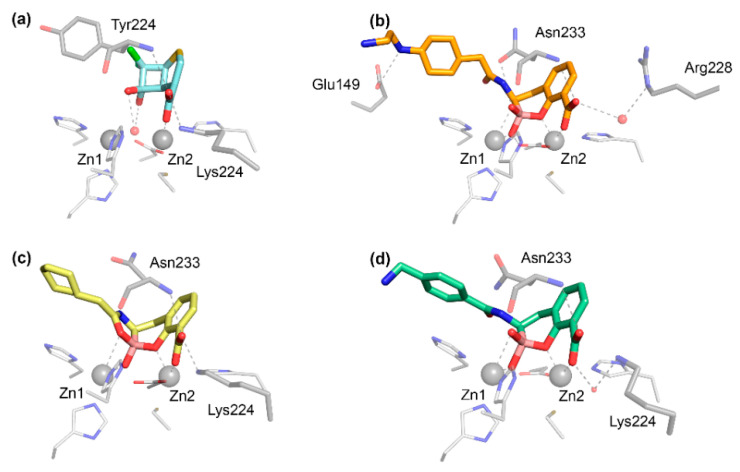
Binding mode of the transition state mimics. Crystal complexes of (**a**) cyclobutanone:SPM-1 (PDB 5ndm) [193], (**b**) Taniborbactam:VIM-2 (PDB 6sp7) [183], (**c**) Taniborbactam:NDM-1 (chain B PDB 6rmf) [162], and (**d**) **14**:BcII (PDB 5fqb) [147]. Hydrogen bonds and metal ligands interactions are drawn with dashed lines.

Transition state analogues are generally efficient enzyme inhibitors, since in principle they are expected to bind more tightly to the enzyme than substrates themselves [186]. In the case of β-lactamases, a first transition state should involve the pre-formation of a tetrahedral carbon giving rise to a stable (in the case of SBLs) or high-energy (MBLs) tetrahedral intermediate. Therefore, transition state analogues inhibitors in β-lactamases attempt to mimic a tetrahedral atom in this position. The synthesis of cyclobutanone analogues was envisaged as a strategy for SBL inhibition based on the premise that the reactive carbonyl group could form a stable hemiketal upon binding to the active site. However, these early attempts were not successful [187,188,189,190,191,192]. More recently, Dmitrienko et al. [156] reconsidered this strategy using cyclobutanones with penem rings. Among the several studied derivatives, cyclobutanone **7** (Figure 6) was identified as a good inhibitor of class A and C SBLs while displaying a weak inhibition of the B1 enzyme IMP-1 (Table 1) [156,193]. X-ray crystallography by the Spencer and Schofield groups succeeded in trapping cyclobutanone **7** bound to the MBL SPM-1 in its hydrated form, as confirmed by Nuclear Magnetic Resonance (NMR) spectroscopy. The bound species presents a tetrahedral C6 with two oxygen atoms, mimicking the transition state generated during β-lactam hydrolysis [156,193]. The C6 oxygen atoms do not form any binding interaction with Zn1 but subtend hydrogen bonds with the preserved bridging water, suggesting that this species is formed in solution and binds the metal site with a tetrahedral carbon atom. Instead, the carboxylate moiety interacts with Zn2, Tyr233, and Lys224 (Figure 7a). The measured affinity (K_D_) of this compound to SPM-1 was 22 µM.

Phosphonate groups have been considered as transition state mimics based on the tetrahedral geometry of the phosphorous atom. This approach has been used to inhibit SBLs [194,195] and, recently several works, MBLs. Mercaptophosphonates **8** (Figure 6) studied by Galleni and Frère were competitive inhibitors of MBLs from the three subclasses (Table 1) [157]. The structures of CphA complexed with some of these compounds did not reveal a common inhibition pattern, since either the thiol moiety or the phosphonate could bind the Zn(II) ion, depending on the inhibitor. None of these structures evidenced that these molecules behaved as transition state mimics [157].

The β-phospholactam **9** (Figure 6) was designed and synthesized by the Yang and Crowder groups. **9** showed a time-dependent inhibition against different B1 and B3 enzymes (Table 1) [158]. As the authors state, it is not clear whether inhibition is due to the phospholactam itself or to its hydrolysis product, that in solution gives rise to a phosphonate. Dmitrienko and Spencer have studied phosphonate-based pyridine-carboxylates **10** (Figure 6) as inhibitors of B1 (VIM-2, NDM-1, and IMP-1) and B3 (L1) enzymes with IC_50_ values in the low-μM range (Table 1). Compound **10** was able to reduce the Minimum Inhibitory Concentration (MIC) of MBL-expressing strains, including *Stenotrophomonas maltophilia*. The structure of the complex of **10** with IMP-1 shows that the carboxylate interacts with Zn2 and Lys224, while the phosphonate moiety interacts with the bridging hydroxide that remains bound to both zinc ions. Binding to L1 occurs upon removal of Zn2 and binding of the phosphonate to Zn1 [159]. At the moment, there is no evidence that phosphonate groups can replace the bridging water in a binuclear enzyme and behave as a transition state mimic.

Boron-based compounds were proposed as analogues of the tetrahedral intermediate of SBLs and the tetrahedral transition state of MBLs based on the ability of boron to mimic either substrates (with a sp^2^ carbonyl group) or the tetrahedral species (with the sp^3^ carbon generated after the nucleophilic attack and before bond cleavage) [111]. This is due to the vacant p orbital of the boron atom that enables the nucleophilic attack. The boron hybridization can be tuned depending on the pKa of the molecule, eliciting a trigonal planar sp^2^ boronic acid or ester, or a tetrahedral sp^3^ boronate anion. In the latter case, the negative charge is mostly localized on the boron atom (while in the transition states, the oxygen atoms bear the negative charge). This feature does not limit the use of boronates and boronic acids as inhibitors of nucleophilic enzymes such as the β-lactamases. The similar lengths of the B–O and C–O bonds as well as the B–C and C–C bonds favor their action as mimics [111].

An acyclic boronic acid with a thiol group was synthesized in an effort to design an inhibitor targeting both MBLs and SBLs [160]. Among different assayed boronic acids, only the thiol-containing compounds were able to inhibit B1 enzymes with *K_i_* values in the low-μM range, although that group was not essential for inhibiting B2 and B3 enzymes. Instead, the boronic acid moiety was essential for SBL inhibition. One of the best fusion-inhibitors **11** (Figure 6) displayed *K_i_* values in the low-μM range against MBLs (Table 1) and class A and C SBLs. **11** was also able to restore the β-lactam efficacy on clinical *Enterobacterales* carrying SBLs [160]. Lastly, a set of acyclic boronates was also described with IC_50_ values in the μM range (20–117 μM) against NDM-1 and VIM-2 also being active towards AmpC, CTX-M-15, and KPC-2 [196].

Cyclic boronates were reported as reversible SBL inhibitors, in which the boron atom is covalently bound to the active site Ser residue after the nucleophilic attack. This is the case with vaborbactam **12** (Figure 6), a monocyclic boronate developed by Rempex Pharmaceuticals (USA) [197] as a potent inhibitor of class A and AmpC enzymes. This compound has been approved by the FDA to treat complicated urinary tract infections in combination with meropenem. However, vaborbactam shows a poor inhibitory activity against MBLs (Table 1) [161].

Bicyclic boronates were recently shown to be promising new scaffolds as MBL inhibitors. Taniborbactam **13** (previously VNRX-5133) (Figure 6), developed by VenatorX Pharmaceuticals (USA) [183,198] is a bicyclic boronate active against MBLs and SBLs. Key to the success of this inhibitor was the introduction of a primary amine instead of a carboxylate group, that dramatically improved the permeability towards Gram-negative bacteria, consistent with the findings of Richter et al. [199]. *K_i_* or IC_50_ values against B1 enzymes cover the nM to the low-μM range (Table 1). However, there are controversial results regarding the inhibition of IMP-1 [162,183,184]. Taniborbactam displayed a moderate inhibition of B2 subclass enzymes, and it was inactive against L1 (Table 1) [162]. The crystal structure of the adduct of taniborbactam with VIM-2 [183] reveals a tetrahedral boron in which one of its oxygen atoms is bound to Zn1, mimicking the transition state (Figure 3b), while the carboxylate interacts with Zn2 and Arg228 (Figure 7b). Instead, taniborbactam complexed with NDM-1 [162] showed two bound species one similar to that reported for VIM-2 and the other including a tricyclic compound (Figure 7c) resulting from an intramolecular coordination of the oxygen of the amide side chain to the boron atom. The latter reaction has been proposed to take place within the active site with the participation of a base, whose identity is unknown. Taniborbactam restored the antibiotic susceptibility of clinical bacterial strains (*Enterobacterales*, *P. aeruginosa*, and *A. baumannii*) [162,183,184]. It did not show toxicity against eukaryotic cells and it was inactive against a panel of human enzymes and receptors. It was also shown to be effective in combination with cefepime in mice infected with *K. pneumoniae*-CTX-M-14 and *E. coli*-CTX-M-15 [183]. This combination is now in phase 3 clinical trials to combat complicated urinary tract infections.

The Schofield group also reported a series of bicyclic boronates. Among them, compound **14** (Figure 6) displayed IC_50_ values against B1 enzymes from the nM to low-μM range (Table 1). Instead, inhibition of CphA was weaker, with a poorer performance than taniborbactam, and the compound was unable to inhibit L1 (Table 1). Compound **14** was also active against Class A and D β-lactamases, with IC_50_ values also in the nM to low-μM range, and at least one PBP. This inhibitor restored the β-lactam efficacy on *K. pneumoniae* and *E. coli* clinical strains carrying MBLs and/or SBLs, with no toxic effects on eukaryotic cells [147]. The adducts with BcII and VIM-2 revealed a similar binding mode than taniborbactam (Figure 7d).

Qpex Biopharma designed the bicyclic boronate QPX7728 **15** (Figure 6), able to inhibit class A ESBLs, the Class C P99, various OXA Class D enzymes, and Class B NDM-1 and VIM-1 (Table 1). The presence of a cyclopropyl group favors hydrophobic interactions in the active site [112,163].

Overall, the use of bicyclic boronates to target all classes of β-lactamases appears highly promising. However, it is not still understood why these compounds are potent inhibitors, while monocyclic boronates are not. More work on this area is required, since mimicking the tetrahedral carbon of a high-energy species seems to provide a common inhibitory strategy targeting both MBLs and SBLs.

### 3.4. Prodrugs and Irreversible Inhibitors

**Figure 8:**
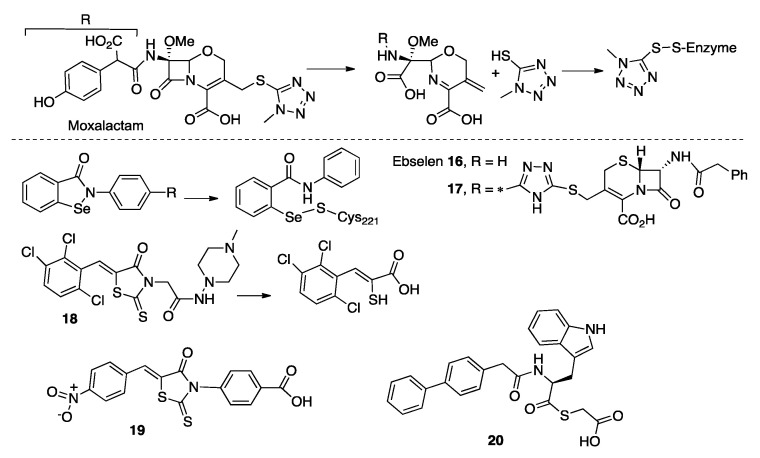
Prodrugs and irreversible compounds proposed as MBL inhibitors.

Some β-lactam antibiotics, upon hydrolysis, give rise to reaction products able to act as MBL inhibitors. The hydrolysis of moxalactam by CphA is followed by elimination of the C3-substituent that releases a 5-mercapto-1-methyltetrazole moiety [200]. This product binds covalently to Cys221, leading to an irreversible inactivation. The same enzyme is also inactivated by the hydrolysis product of cefoxitin, although the mechanism is not still clarified. It is likely that this inhibition results from the formation of a disulfide bond between Cys221 and the sulfur of the six-membered ring of the product (Figure 8) [200].

The hydrolysis of some cephalosporins by BcII had also led to product inhibition. After β-lactam bond cleavage, the six-membered ring is opened due to C6 epimerization, with cleavage of the C-S bond thus leaving a free the thiol group that binds the Zn(II) ions [201]. These results could be exploited to inspire the design of prodrugs/suicide MBLs inhibitors that release the active drug in the enzyme active site.

Ebselen/SPI-1005 **16** (Figure 8), is a selenium-based MBL inhibitor [202] initially identified as a substrate mimic of glutathione peroxidase [203]. The compound is now in clinical trials for different diseases (Meniere disease, tobramycin induced ototoxicity and to prevent acute noise induced hearing loss). Ebselen inhibited B1 and B2 enzymes with IC_50_ values in the low-μM range but was not active against the B3 subclass (Table 1) [164]. The inhibitor acts by forming a covalent bond between the Se atom and Cys221 coupled to dissociation of the Zn(II) ion at site 2 [202]. The scaffold was modified by fragment modification or rationally adding an activated ester targeting Lys224 but, as a result, the IC_50_ values were similar to those from the unmodified compound [164,165]. Aiming to target L1 β-lactamase (lacking the Cys ligand), the Ebselen scaffold was attached to a cephalosporin moiety. The resulting compound **17** (Figure 8) displayed low-μM IC_50_ values towards the three subclasses (Table 1). All the assessed compounds could effectively restore the antibacterial efficacy of β-lactam antibiotics on laboratory strains of *E. coli* harboring MBLs and in a clinical *E. coli* variant expressing NDM-1 [164,165,202]. Similar results were obtained with Ebsulfur, an NDM-1 inhibitor in which the Se was substituted by a sulfur atom [204].

Rhodanines were initially identified as class A and C [205] and PBP [206] inhibitors by high-throughput screenings. Compound ML302 **18** (Figure 8) was later studied by Schofield et al. as an inhibitor of B1 MBLs with IC_50_ values in the low-μM range (Table 1) [207]. The inhibitory action resides in the release of a thiol group after hydrolysis of the rhodanine by the MBLs, which results in inhibition by the thiol moiety bridging the two Zn(II) ions. The intact rhodanine molecule could also be found bound to the active site. This compound was effective in reducing the MICs toward meropenem on clinical *Enterobacterales* expressing B1 enzymes. The compound was not toxic against eukaryotic cell lines, but showed cross inhibition of the human angiotensin-converting enzyme (ACE) [166]. More rhodanine analogues were synthetized and analyzed as broad-spectrum MBL inhibitors, with IC_50_ values in the low-μM range. The performance of the most potent inhibitor of this series, **19** (Figure 8), is reported in Table 1. Since one of the assessed rhodanine analogues was not hydrolyzed by L1, the authors proposed that the intact compound is responsible for inhibition. Its thioenolate product, obtained by chemical hydrolysis, was only able to inhibit L1 and VIM-2 but was inactive against NDM-1 or ImiS. Compound **19** restored the antimicrobial activity of β-lactams in laboratory strains of *E. coli* expressing different MBLs and no toxicity was observed against eukaryotic cells [167]. Finally, another set of **18** derivatives and their products, the hydrolyzed enethiol, acted as MBL inhibitors [208]. The different binding modes and activities observed for rhodanine derivatives require further investigations.

Amino-acid-based thioesters such as **20** (Figure 8) were reported as inhibitors of L1, NDM-1 and ImiS [209]. This scaffold was later optimized to specifically target L1 [210]. The inhibition mode was characterized by Chen et al., who found that one third of the thioesters were hydrolyzed during the experiments and both, the compound and the product, contributed to inhibition [211]. A new series of inhibitors of this family present substrate-mimicking features and undergo hydrolysis, releasing inhibitory compounds, likely thioglycolic acid **20** (Figure 8 and Table 1) [168].

## 4. Intermediate Species and Enzyme: Product Complexes as Templates for Minimalistic Scaffolds

### 4.1. Intermediate Species Formed during MBL Mechanism

The identification of mechanistic intermediates in MBL-mediated catalysis has been initially pursued by the use of chromogenic derivatives of cephalosporins, such as nitrocefin and chromacef. These compounds are spectroscopic probes of the reaction mechanism because of their spectroscopic features (not present in clinical substrates) [88,89,100,102,129,212,213,214,215]. Nitrocefin and chromacef hydrolysis take place with the accumulation of an anionic reaction intermediate (Figure 3b,c). The negative charge of this species is stabilized by a direct interaction with Zn2 and is delocalized in a conjugated π system present in the chromophore (Figure 3c). Unlike the tetrahedral transition state and the reaction intermediate of SBLs, the C-N bond of the β-lactam ring is broken in this intermediate and the β-lactam carbonyl is converted into a carboxylate moiety (Figure 3). The generalization of these findings to other β-lactams was limited during the last couple of decades because (1) the stabilization of the anionic intermediate was considered an artifact due to the particular nature of the chromophore present in these compounds and (2) these cephalosporins could not be used as probes of the mechanism on B2 enzymes, since they are exclusive carbapenemases.

Mechanistic studies [86,87,100,101,102,103,145], X-ray crystallography [53,144,146,216], and computational studies [87,104,105,106,217] have supported the accumulation and stability of anionic intermediates on the three MBL subclasses with clinical cephalosporins and carbapenems. Instead, no reaction intermediates have been observed during penicillin hydrolysis [131], a fact that can be attributed to the presence of a saturated five-membered ring in these substrates that may preclude the accumulation of the anionic species. 

X-ray crystallography has been employed to trap a cephalosporoate intermediate in the hydrolysis of clinical cephalosporins by NDM-1 [101], supporting the evidence from the spectroscopic studies with nitrocefin [100]. Computational studies also verified the stability of anionic reaction intermediates during cephalexin hydrolysis, whose negative charges are stabilized by the interaction with Zn2 [104]. For cephalosporins containing good leaving groups, it has been proposed that R^1^ is eliminated without evidence of intermediate formation [216].

Accumulation of the intermediate during carbapenem hydrolysis has been confirmed by rapid kinetics studies [86,87,102,103]. This reaction courses by a branched mechanism that includes the same two productive intermediate species, valid for MBLs of the three subclasses (Figure 3c) [87]. These are anionic intermediates in which the C-N bond is already broken and resemble the species accumulated during cephalosporin hydrolysis (Figure 3b,c) [86]. In both cases, the hydrolyzed and deprotonated carbapenem interacts with Zn1 through the C7 carboxylate and with Zn2 through the N atom and the C3 carboxylate (Figure 3c). Both in mono- and bi-metallated MBLs, the negative charge is stabilized by interaction with Zn2, highlighting the functional relevance of this metal site [87]. The stability of the negative charge on these species was also supported by computational analysis [87,105,106].

The two anionic species described differ on the proton donor that gives rise to product formation [87]. These could correspond to a metal-activated water molecule that protonates the N atom or a bulk water molecule able to protonate the C3. Depending on the proton donor, two different products can be formed during enzyme hydrolysis, as shown by NMR [86,87,218]. As in the case of nitrocefin derivatives [100], the anionic species are quite stable, therefore, the protonation step giving rise to the product is rate-limiting [86,87,216]. The stability of the reaction intermediates is variable depending on the enzyme and substrate since it is determined by the interactions with residues near the enzyme active site [70,89,219,220], active site loops [60,65], and metal ions [87,221].

### 4.2. MBL: Product Complexes

**Figure 9:**
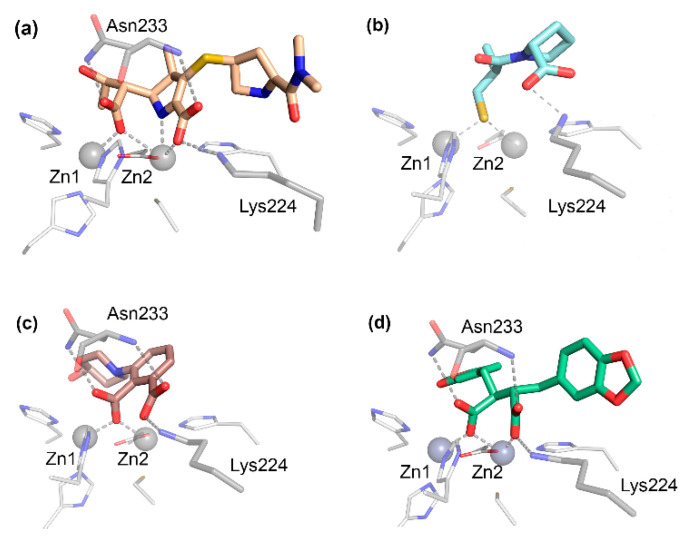
Binding modes of the product and intermediate mimic inhibitors. Crystal complexes of (**a**) NDM-1:meropenem hydrolysis product (PDB 4eyl) [69], (**b**) IMP-1:D-captopril (PDB 4c1g) [169], (**c**) a 3-aminophtalic acid inhibitor with IMP-1 (PDB 3wxc) [226], and (**d**) IMP-1:**24** (PDB 1jjt) [173]. Hydrogen bonds and metal ligands interactions are drawn with dashed lines.

The last step in the mechanism is the protonation of the anionic intermediate, which leads to the formation of the EP complex (Figure 3b and Figure 9a) [87,88,89]. EP can be a transient complex rapidly leading to product release and recovery of the free enzyme or, depending on the affinity of the adduct, it can give rise to product inhibition. Several crystal structures of MBLs with hydrolyzed substrates have been reported. It is important to highlight that these binding modes may not strictly correspond to the EP complexes, since in some cases the adducts were obtained by addition of the hydrolyzed products to the enzyme, and even when the reaction takes place in crystallo, the products can be released from the enzyme and rebound later.

The first structure of this series corresponds to CphA bound to hydrolyzed biapenem [53]. In this case, the product binds through a direct carboxylate-Zn(II) coordinative bond, supported by secondary interactions with Lys224 and Val67. The first reported EP crystal structure for a bi-metallated MBL was that of L1 bound to hydrolyzed moxalactam, a clinically used cephalosporin antibiotic [138]. This structure disclosed for the first time a direct interaction of Zn2 in a binuclear enzyme with the N atom and the C4 carboxylate of cephalosporins after bond cleavage, as well as the binding of the C8 carboxylate in the product with Zn1. Information about product binding on B1 enzymes dates back to 2011 with a series of structures of NDM-1 bound to different hydrolyzed substrates [69,101,135,139,216,222,223]. All these structures show a similar interaction of the β-lactam ring with the active site, but with the R^1^ groups adopting different conformations. The hydrolyzed β-lactam group gives rise to a carboxylate that, together with the conserved carboxylate from the substrates, coordinate the metal ions of the active site (Figure 9a). The N atom and C3 carboxylate bind the Zn2, while the C7 carboxylate of hydrolyzed substrates can be bound to Zn1 or can bridge both metal ions. Further interactions are the C7 carboxylate with Asn233 and the C3 carboxylate with Asn233, Lys224, and Val67. Enzymes from the VIM family lack Lys224, and the carboxylate oxygen bound to Zn2 interacts with an extra water molecule and backbone atoms from Cys221 [140]. The different substituents are located on the grooves defined by the active site L3 and L10, but few specific interactions have been reported [101,134,139].

The nature of the proton donor has been a matter of intense debate. Asp120 was originally proposed as the proton donor due to its essentiality for the enzyme activity [77,83,224]. Today, a water molecule is the most accepted proton donor, but there is some controversy about the nature of this molecule [53,74,83,87,88,89,105,106,135,144]. The available structures of the EP complexes do not provide a consensus solution to this conundrum. For example, structures with hydrolyzed carbapenems [69,216] lack a bridging water that is present in structures with hydrolyzed penicillins and cephalosporins [69,101,135,139,216], suggesting that the protonation process depends on the substrate. The presence of that water molecule depends on the binding mode of the C7 carboxylate [69]. Quantum mechanics/molecular mechanics (QM/MM) studies suggest that an EP complex with a carbapenem is stable only in the absence of a metal bridging ligand [87]. Otherwise, in the presence of a water molecule, it is predicted that product release will take place rapidly [87]. These calculations account for the fact that crystallized EP complexes with carbapenems lack a bridging water/hydroxide molecule [69,216]. 

A bridging water molecule would be able to protonate the N atom. Nevertheless, for carbapenem hydrolysis it has been demonstrated that the protonation event can occur both in the N or C3 atoms, as a consequence of a branched mechanism with two productive reaction intermediates. The ratio between the different products was studied by NMR spectroscopy by several groups [86,87,216,218], indicating that the main product of carbapenem hydrolysis results from protonation of the N atom. There are controversial results regarding the stereochemistry of C3 protonation [87,216,218]. Crystallographic structures have revealed EP complexes protonated only at the β face of the antibiotic [216,225], and different NMR analysis have proposed the predominance of both species [87,216,218]. Hydrolysis of cephalosporins with poor leaving groups give rise to protonation on the α face [101]. This proposal has also been analyzed by computational studies and X-ray crystallography, concluding that the identity of the proton donor depends on the substrate [104,216].

### 4.3. Inhibitors Based on EI/EP Structures

The enzyme-bound EI and EP species have two features in common (1) the β-lactam bond has already been cleaved and (2) the presence of two carboxylate groups binding the Zn(II) ions (Figure 3). As these species form stable and conserved interactions with the enzyme, both criteria have been considered for the choice of known molecules and the design of new compounds as intermediate/product mimics. 

**Figure 10:**
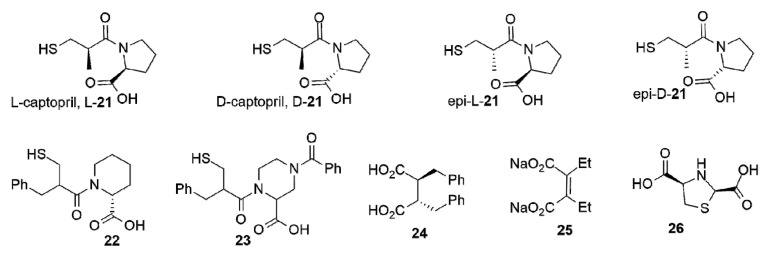
Intermediate and product mimic compounds studied as MBL inhibitors.

L-captopril L-**21** (Figure 10) is an inhibitor of the ACE usually prescribed to relieve hypertension [227]. Its structure partially resembles some features of hydrolyzed penicillins. L-captopril was explored early on as an inhibitor of BcII and CphA. Both L- and D-enantiomers inhibited BcII within the μM range. Instead, the stereochemistry played a key role in the inhibition of CphA, with D-captopril performing 10-fold better than the L stereoisomer (Table 1). The binding modes were analyzed by different spectroscopic techniques [170]. The first crystal structures of adducts with captopril that were solved were those of BlaB [228] and FEZ-1 [229] with D-captopril and then with L1 [171], CphA [230] (with the same enantiomer), and NDM-1:L-captopril [69]. Later, the following four captopril stereoisomers were analyzed: L-captopril L-**21**, D-captopril D-**21**, epi-L-captopril epi-L-**21**, and epi-D-captopril epi-D-**21** (Figure 10) [169]. The D-enantiomer showed the highest potency against a panel of different MBLs, while the diasteroisomers were poor MBL inhibitors (Table 1). The binding modes were analyzed obtaining the crystal structures of VIM-2, IMP-1 and BcII in complex with D- and L-captopril [169]. The thiol group bridging both Zn(II) ions is the driving force for inhibition, while the carboxylate group forms different interactions depending on the enzyme and the stereoisomer (Figure 9b). The most potent inhibitors displayed a higher number of hydrogen bonds and interactions with the conserved Lys224 or Arg228 (VIM enzymes) also involved in substrate binding. These compounds were also stabilized through hydrophobic contacts with L3 and hydrogen bonds with L10 [169]. Removal of the methyl group or a different length of the thioalkyl side chain led to minor changes on the inhibition potency, but lengthening the tether for the carboxylate group decreased the activity on IMP-1 [231]. Büttner et al. also examined different captopril analogues, and the substitution of the methyl group by a phenyl ring led to similar or reduced potencies on NDM-1, IMP-7, and VIM-2 [231].

The use of analogues with a six-membered pyrrolidine ring did not impact on the activity of captopril and the essentiality of the thiol and the carboxylate functionalities were also explored in both compounds [231]. Other six-membered ring analogues that were assessed are the piperidine **22** and piperazine rings **23** (Figure 10), that can be considered as product/intermediate mimics with a cepham ring, leading to inhibition within the low-µM range (Table 1) [231].

Among different inhibitors with two carboxylate groups, a succinic acid derivative was found in a screening of a chemical collection from Merck Chemical Co [173]. The derivative **24** (Figure 10) bearing aromatic substituents was an IMP-1 inhibitor in the nM range (Table 1). One carboxylate group binds Zn2 and interacts with Lys224 and the side chain of Asn233, while the second carboxylate bridges the two Zn(II) ions, replacing the water molecule, and mimicking some of the aspects observed in several enzyme-product complexes (Figure 9d) [173]. A similar binding mode was observed for the 3-aminophtalic acid inhibitor and IMP-1, which showed an IC_50_ value of 2.51 µM (Figure 9c) [226]. Maleic acid **25** (Figure 10) is a simple scaffold that showed low-µM or µM inhibition against some B1 enzymes (Table 1) and was able to restore the carbapenem efficacy on MBL-producing *P. aeruginosa* [174,185,232]. N-heterocyclic dicarboxylic acids were characterized as MBL inhibitors. Within this series, thiazolidine **26** (Figure 10) shows low-µM *K_i_* values against the three subclasses (Table 1) and was also able to restore the β-lactam potency on *E. coli* laboratory strains expressing the same β-lactamases [175].

## 5. Zn(II) Displacement as a Strategy for MBL Inhibition

MBLs, being Zn(II)-dependent enzymes, are sensitive to the presence of metal chelators. Indeed, Zn(II) removal by chelating agents is a common strategy in biochemical studies of MBLs to probe the role of the metal cofactors, usually complemented by metal substitution [46,99,131,233,234,235,236]. The presence of chelating agents in microbiology assays is also widely used as a phenotypic test to identify the presence of an active MBL in bacteria. This strategy is employed by the commercial Etest (bioMérieux, Marcy L’Etoile, France), the Total Metallo-β-lactamase Confirm Kit (ROSCO, Taastrup, Denmark) and in the reported assays by Marchiaro et al. (EIM test) [237], Sfeir et al. (eCIM test) [238], and Giske et al. [239]. Despite the efficacy of chelators in vitro, their use as inhibitors is controversial based in their low specificity that may target many other metalloproteins.

**Figure 11:**
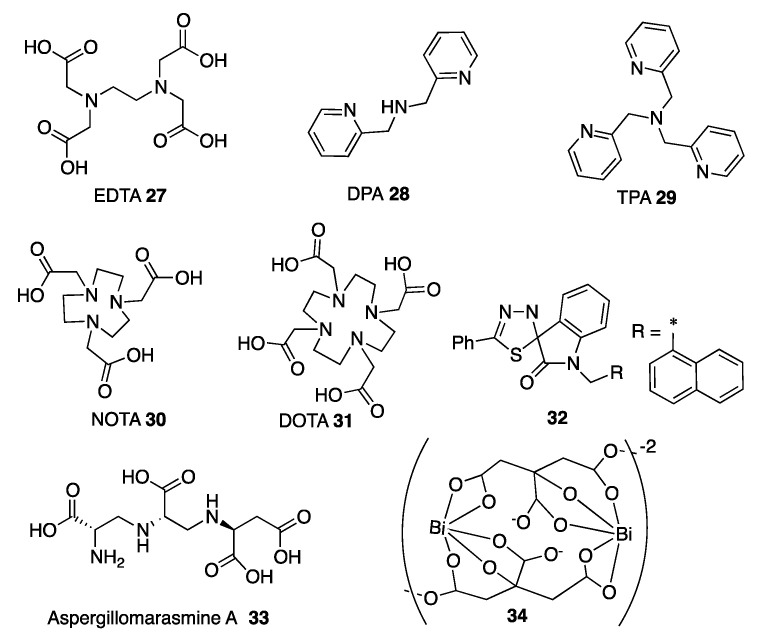
Chelators being studied as MBL inhibitors.

One of the first metal chelators described as an MBL inhibitor is the well-known metal sequestering agent ethylenediaminetetraacetic acid (EDTA) **27** (Figure 11). EDTA restores the efficacy of β-lactams in bacterial pathogens producing MBLs and also presents antimicrobial effectiveness by itself [240]. Despite its activity in bacteria, the cytotoxic effects against eukaryotic cells prevent its therapeutic use [241,242]. The only approved treatment with EDTA is the calcium disodium form to treat lead poisoning. This combination potentiated the β-lactam activity against *P. aeruginosa* isolates producing IMP or VIM β-lactamases and was also effective in mice infected with carbapenem-resistant *P. aeruginosa* [243] and *E. coli* producing NDM-1 [244].

Other chelating agents assayed as MBL inhibitors are different picolinic acid derivatives such as dipicolinic acid (DPA) **28**, tris(2-pyridylmethyl)amine (TPA) **29**, and N,N,N’,N’-tetrakis(2-pyridylmethyl)ethylenediamine (TPEN). They restored the sensitivity to meropenem of *Enterobacterales* producing NDM or VIM β-lactamases; in the case of IMP-8, only TPA and TPEN were shown to be active [242,245]. Regarding the toxicity, DPA showed reduction of viable eukaryotic cells [242].

Cyclam-based chelating agents such as 2,2′,2″-(1,4,7-triazacyclononane-1,4,7-triyl)triacetic acid (NOTA) **30** and 1,4,7,10-tetraazacyclododecane-1,4,7,10-tetraacetic acid (DOTA) **31** (Figure 11) were active against different carbapenem-resistant bacteria expressing NDM, VIM or IMP enzymes [246]. As NOTA was the most active compound, different analogues were synthetized and some of them (**30** is an example) were able to restore the activity of meropenem toward carbapenem-resistant pathogens without showing toxicity against eukaryotic cells [247]. A related compound, 1,4,7-triazacyclononane (TACN), in combination with meropenem, showed bactericidal effects on different clinical *Enterobacterales* expressing B1 MBLs but it was shown to compromise the eukaryotic cell viability [248].

The spiro-indoline-thiadiazole compound (SIT-Z5) **32** is a specific Zn(II) chelator and was shown to perturb the metal ion homeostasis in *E. coli*. The compound also restored the activity of meropenem against a *K. pneumoniae* strain expressing NDM-1 in vitro and in infected mice. It showed an IC_50_ value with NDM-1 of 6.6 μM, while it was unable to inhibit IMP or class A β-lactamases [249]. 

Aspergillomarasmine A (AMA) **33** (Figure 11) is a natural product isolated from the extract of the fermentation broth of *Aspergillus versicolor.* AMA had been characterized in 1965 [117,250,251], but it was only recently identified as a specific MBL inhibitor [179]. This compound displayed low-µM IC_50_ values for NDM-1 and VIM-2 [180,181], and above 500 μM against IMP-7 (Table 1) [179]. The activity of AMA and some synthetic isomers and analogues was analyzed against different B1 MBLs, but none of these compounds performed better than the natural product [180,181]. AMA restored meropenem efficacy against clinical strains of *Enterobacterales*, *A. baumannii* and *P. aeruginosa* producing NDM or VIM, but was inactive in the presence of SPM-1, AIM or IMP, consistent with its high IC_50_ values. The effect of AMA was shown to be dependent on the MBL and the antibiotic partner, possibly due to the affinity of each enzyme towards Zn(II) ions [252]. Lastly, the compound was not toxic to mice and eukaryotic cells, and in combination with meropenem was effective in mice infected with *K. pneumoniae* NDM-1 producers [179,253].

Finally, bismuth compounds were recently recognized as MBL inhibitors [182]. In particular, colloidal bismuth subcitrate **34** (Figure 11), an approved drug for the treatment of *H. pylori*, potentiated meropenem activity against some clinical *Enterobacterales* producing NDM-1 and *E. coli* BL21 cells expressing VIM or IMP β-lactamases. **34** showed comparable IC_50_ values for NDM-1, VIM-2, and IMP-4 in the low-µM range (Table 1). The rationale for the action of Bi(III) is based on its ability to displace both Zn(II) ions from the metal site. Indeed, Bi(III)-inhibited NDM-1 harbors one equivalent of this metal ion bound to ligands from the Zn1 and Zn2 sites (Cys221, Asp120, His116, and His196 and a water molecule). This leads to an irreversible MBL inhibition, since addition of Zn(II) ion cannot revert this effect on the Bi(III)-substituted enzyme. The ability of the inhibitor to restore meropenem efficacy was confirmed in a mouse infection model [182].

Metal sequestration is a natural defense mechanism in vertebrate hosts against bacterial infections, in which the host sequesters transition metal ions to limit their availability as they are essential nutrients of bacteria [254,255]. Among different proteins being secreted to achieve metal starvation, calprotectin (CP) sequesters divalent transition metal ions, in particular Zn(II) [256]. As a result, when MBLs are expressed, this immune response inactivates the β-lactamases by removing their essential metal ions in the periplasmic space, resulting in MBL degradation [257]. On one hand, the use of chelators mimics this natural strategy. On the other hand, the exposure to this metal starvation environment may have led to the selection of MBLs that have developed a higher Zn(II) binding affinity. Indeed, the analysis of current NDM alleles showed that these enzymes are under the evolutionary pressure of Zn(II) limitation and could escape the action of chelators by incorporating mutations that increase the Zn(II) binding affinity [258,259]. These two aspects should be considered when evaluating the use of metal chelators to combat MBLs.

## 6. Perspectives and Concluding Remarks

During the early years of the MBL research, the substrate structures were extensively explored as templates for inhibitors due to the lack of crystal structures. With the availability of new techniques such as libraries and fragment-based screenings, many compounds were reported with no similarities with the catalytic species. Most of them were active only against a few MBLs belonging to the same subclass. Later, the elucidation of the catalytic mechanism opened new paths for the design of novel compounds. The transition state and intermediate species were employed as templates to develop inhibitors with cross class activities. In this regard, boron-based compounds represent some of the most promising compounds, since they can achieve inhibition of all β-lactamases classes by mimicking common features of the tetrahedral intermediate of SBLs and the tetrahedral transition state in MBLs. It is very likely that the first approved MBL inhibitor will indeed bear a boronate (taniborbactam). However, there are still many issues to be explored in this field, such as understanding why the monocyclic boronate vaborbactam is inactive against MBLs. 

Thiol-based compounds are also an interesting scaffold for MBL inhibition, but further improvements are necessary due to its sensitivity to oxidation. The development of prodrugs in which the thiol group is protected and can be released within the cell is a possible strategy to exploit the avidity of thiol moieties towards Zn(II). 

Finally, Zn(II) displacement is an increasingly appealing strategy. Among all studied compounds, AMA and bismuth are the most promising agents. However, this approach might be questionable due to the low specify of the metal chelators and also by considering that the use of chelators as inhibitors may exert an evolutionary pressure to enhance the Zn(II) binding affinities of MBLs. 

## Figures and Tables

**Table 1:** Inhibition potency against MBLs, IC_50_, or *K_i_* (μM).

Inh	B1					B2			B3		Ref
	NDM	IMP	VIM	BcII	Other	ImiS	CphA	Sfh-I	L1	GOB	
Substrate mimic											
**1**	-	0.09	-	-	-	-	-	-	0.5	-	[148]
**2**	-	0.004 *	-	0.83 *	0.23 ^1,^*	-	-	-	1 *	-	[151]
**3**	-	-	2	-	0.3 ^2^	-	-	-	-	-	[152]
**4**	-	-	-	1.4	-	-	-	-	0.1	-	[153]
**5a**	-	720 *	-	-	-	-	-	-	-	-	[154]
**L-6**	7 *	8 *	2.9 *	36 *	-	-	-	0.26 *	12 *	41 *	[149,150]
Transition state analogues											
**7**	-	213	-	-	-	-	-	-	-	-	[156]
**8**	-	-	4 *	-	-	-	5 *	-	0.40 *	-	[157]
**9**	(53)	(70)	-	Act ^3^	(70) ^1^	-	-	Act ^4^	(70)	-	[158]
**10**	0.37	3.88	1.29	-	-	-	-	-	1.48	-	[159]
**11**	6.06 *	-	0.07 *	-	-	-	-	0.72 *	-	10.98 *	[160]
**12**	631	126	316	-	-	-	631	-	336	-	[161]
**13 ^5^**	0.01	2.51	0.0005	-	-	-	2.51	-	>10	-	[162]
**14**	0.03	1	0.003	0.3	16.7 ^3^	-	>100	-	N.I.	-	[147]
**15**	0.032 *	0.008 *	0.22 *	-	-	-	-	-	N.I.	-	[163]
Prodrug and Irreversible inhibitors											
**16**	0.072	1.59	-	-	-	9.56	-	-	N.I.	-	[164]
**17**	2.93	0.28	-	-	-	4.86	-	-	6.68	-	[165]
**18**	9.44	0.17	0.66	0.50	1.32 ^3^	-	-	-	-	-	[166]
**19**	1.31	-	0.19	-	-	3.0	-	-	-	0.05	[167]
**20**	16	12	-	-	-	0.02	-	-	0.08	-	[168]
Intermediate or product mimic											
**L-** **21**	157	23	4.4	80.4	> 500 ^3^	-	950 *	-	20	-	[169,170,171]
**D-** **21**	20.1	7.2	0.072	10.7	261 ^3^	-	72 *	-	-	-	[169,170]
**L epi-** **21**	> 500	436	5.5	424	> 500 ^3^	-	-	-	-	-	[169]
**D epi-** **21**	65	173	5.5	>500	> 500^3^	-	-	-	-	-	[169]
**22**	0.7	0.9	4.6	-	-	-	-	-	-	-	[172]
**23**	2.5	0.3	4.5	-	-	-	-	-	-	-	[172]
**24**	-	0.0027	-	-	-	-	-	-	-	-	[173]
**25**	24 *	1 *	0.46 ^6,^*	-		-	-	-	-	-	[174]
**26**	-	-	-	-	0.64 ^1,^*	7.1 *	-	-	1.8 *	-	[175]
Metal displacement agents											
**27**	0.41	27.9	9.3	-	175 ^7^	-	-	-	-	-	[176,177]
**28**	0.41	3.03	1.66	-	-	-	-	-	-	-	[178]
**33**	11	(70)	7	-	-	-	-	-	-	-	[179,180,181]
**34**	2.81	0.70	3.55	-	-	-	-	-	-	-	[182]

The values with an asterisk * are *K_i_* determinations. The values between parentheses represents the inhibition percentage at the assessed concentration. ^1^ CcrA; ^2^ GIM-1; ^3^ SPM-1; ^4^ The compound activates Sfh-I; ^5^ additional determinations: IC_50_ NDM-1 0.19 μM, VIM-2 0.026 μM, and IMP-1 39.8 μM [183] and *K_i_* NDM-1 0.081 μM, IMP-1 >30 μM, and VIM-2 0.019 μM [184]; ^6^ Additional determinations: *K_i_* of 120 μM [185]; ^7^ DIM-1.
